# Mutational robustness and the role of buffer genes in evolvability

**DOI:** 10.1038/s44318-024-00109-1

**Published:** 2024-05-08

**Authors:** Mohammed T Tawfeeq, Karin Voordeckers, Pieter van den Berg, Sander K Govers, Jan Michiels, Kevin J Verstrepen

**Affiliations:** 1 VIB-KU Leuven Center for Microbiology, Leuven, Belgium; 2 Department of Microbial and Molecular Systems, KU Leuven, Leuven, Belgium; 3 Department of Biology, KU Leuven, Leuven, Belgium

**Keywords:** Cryptic Genetic Variation, Evolvability, Genetic Buffering, Hsp90, Mutational Robustness, Evolution & Ecology

## Abstract

Organisms rely on mutations to fuel adaptive evolution. However, many mutations impose a negative effect on fitness. Cells may have therefore evolved mechanisms that affect the phenotypic effects of mutations, thus conferring mutational robustness. Specifically, so-called buffer genes are hypothesized to interact directly or indirectly with genetic variation and reduce its effect on fitness. Environmental or genetic perturbations can change the interaction between buffer genes and genetic variation, thereby unmasking the genetic variation’s phenotypic effects and thus providing a source of variation for natural selection to act on. This review provides an overview of our understanding of mutational robustness and buffer genes, with the chaperone gene *HSP90* as a key example. It discusses whether buffer genes merely affect standing variation or also interact with de novo mutations, how mutational robustness could influence evolution, and whether mutational robustness might be an evolved trait or rather a mere side-effect of complex genetic interactions.

## Abstract (web summary)

This review uses the example of the chaperone gene HSP90 to outline our current understanding of mutational robustness and the role of buffer genes in evolution.

## Graphical abstract

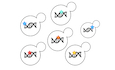

## Introduction

Mutations are essential for organisms to adapt to novel or changing environments and are therefore at the very heart of evolution. However, although mutations can be beneficial, many are neutral or have a negative fitness effect (Eyre-Walker and Keightley, 2007; Perfeito et al, 2007; Elena and Lenski, 2003; Levy et al, 2015). Consequently, one of the main conundrums in biology revolves around the paradoxical vital role of mutations as a source for adaptive evolution and their potentially destructive effects in the evolutionary race. Early research suggested that cells have evolved in such a way that the average genomic mutation rates fall roughly within the same order of magnitude across the tree of life (Drake, 1999, 1991). More recent work suggests that the average genomic mutation rate might be more variable (Lynch, 2010; Gorelick and Naxerova, 2022; Bergeron et al, 2023; Lynch et al, 2023). Nevertheless, it is generally accepted that evolution itself has selected genomic mutation rates that are low enough to avoid rapid extinction due to the accumulation of detrimental mutations, but not so low that the underlying molecular machinery would impose an excessive physiological burden or block evolutionary innovation. However, the optimal mutation rate depends on many factors, including the ability for sexual reproduction, multicellularity, population size, selective pressure, and the cellular cost of the mechanisms that allow tuning of mutation rates, such as proofreading and DNA repair (Rando and Verstrepen, 2007; Denamur and Matic, 2006; Sniegowski et al, 1997).

The main processes that are responsible for the tuning of mutation rates include DNA replication and repair (Kunkel and Bebenek, 2000; Lindahl, 1993; Sniegowski et al, 1997; Modrich, 2006; Lang and Murray, 2011). However, less obvious phenomena may also play a role. For example, mutation rates are not equal across the genome and it has been suggested that genomes have evolved in such a way that mutations occur more frequently in genes where they are more likely to be adaptive, such as genes encoding outer cell surface proteins (Rando and Verstrepen, 2007). However, this is not to suggest that mutation rates are actively tuned towards genes/loci where they could be adaptive. Instead, genes or loci where mutations are more likely to be adaptive have been selected to be located in genomic regions that show higher mutation rates such as the sub-telomeres, or they may contain unstable genetic elements, such as tandem repeats (Gemayel et al, 2010; Brown et al, 2010). Similarly, mutation rates are sometimes higher in stressful environments, when the need for adaptation is higher (Shor et al, 2013; Foster, 2007; Pribis et al, 2022; Bjedov et al, 2003; Swings et al, 2017). In some cases, such mechanisms are clearly the result of direct selection, but in other cases, this is less clear (Rando and Verstrepen, 2007).

Importantly, most mutations only have a limited fitness effect (Levy et al, 2015). In 1942, Waddington proposed that developmental processes are quite robust and result in the same phenotype regardless of minor genetic variation, which he referred to as canalization (Waddington, 1942). Waddington believed that this robustness was not solely a consequence of near-neutral mutations but instead hypothesized a more complex cellular property. This property, under certain conditions, could be lost, resulting in the phenotypic exposure of the previously neutral genetic variation and perhaps providing a substrate for natural selection. Later, it became clear that some of the effects that Waddington referred to might be attributed to complex genetic interactions, where the effect of a mutation is influenced by the presence or absence of other mutations or alleles. One specific example is suppressor mutations (i.e., secondary mutations that help restore fitness after the occurrence of a primary mutation with a negative fitness effect). Such interactions lead to complex genetic patterns and help contribute to our understanding of how mutations can interact to shape phenotypic outcomes (Siegal and Bergman, 2002; Rutherford and Lindquist, 1998; Wagner, 2000b).

**Figure 1:**
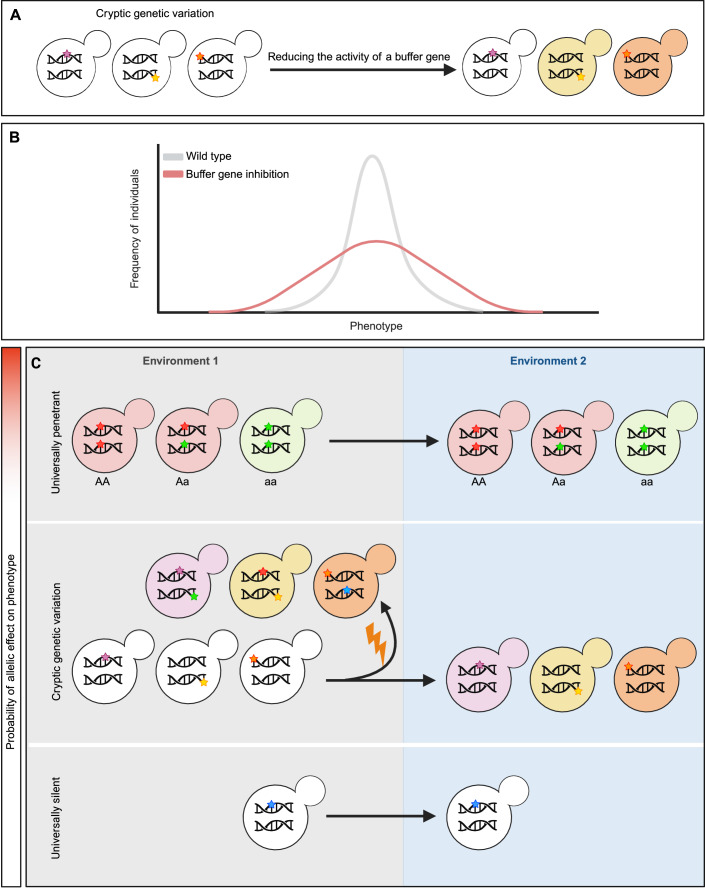
Buffer genes influence phenotypic variation. (**A**) The concept of mutational robustness via buffer genes: simplification of the concept of mutational robustness and how reducing the activity of one gene could reveal phenotypic effects of genetic variation. Colored stars in the yeast cell’s DNA represent mutations and the different yeast colors represent phenotypically active genetic variation. (**B**) Buffer genes and phenotypic diversity: inhibiting a gene that buffers the phenotypic effects of genetic variation will increase phenotypic diversity in a population. (**C**) Simplification of standing genetic variation categories: On one extreme, alleles that are universally penetrant for the three genotypes at a locus—*AA* (homozygous dominant), *Aa* (heterozygous), and *aa* (homozygous recessive)—consistently yield the same phenotype across various environments. On the other extreme, universally silent alleles do not produce any phenotype in any environment. Cryptic genetic variation is a subcategory of standing genetic variation that only exhibits a phenotype in the presence of perturbations, such as environmental or genetic changes, that interact with the cryptic genetic variation. The lightning symbol represents factors that induce genetic changes, such as radiation, chemicals, or biological processes that cause mutations. The color gradient in this figure is designed to represent the probability that a specific allele will influence the observable traits, or phenotype, of an organism. The gradient transitions from white to red, where white signifies a lower probability of the allelic effect, indicating minimal or no impact of the allele on the phenotype. Color intensification towards red indicates an increasing probability of the allelic effect. Please note that this complete figure is conceptual, and the colors and patterns used in the figure do not represent specific mutations, genes, or phenotypic effects observed in experiments. Figure created with BioRender.com.

A special case of genetic interaction has been uncovered in seminal studies showing that the activity of a specific gene product can alter the phenotypic effects of genetic variation elsewhere in the genome (Queitsch et al, 2002; Jarosz and Lindquist, 2010; Rutherford and Lindquist, 1998). Consequently, reducing the activity of such a “buffer gene” changes the phenotypic effect of some standing genetic variation (i.e., genetic variation present within a population that has been subjected to natural selection) (Fig. 1A–C). In other words, previously hidden genetic variation (so-called cryptic) can be revealed. Intriguingly, it has been suggested that buffer genes might also mitigate the phenotypic outcome of (a subset of) de novo mutations (i.e., new genetic variation that has not yet experienced natural selection) and thus can affect the capacity of the population to adapt to new selective challenges in several ways (Geiler-Samerotte et al, 2019, 2016). However, only very few studies investigate the buffering of de novo mutations and the existence of genes that buffer de novo mutations is contested (Richardson et al, 2013; Geiler-Samerotte et al, 2016). In addition, the underlying molecular mechanisms also remain largely elusive. Moreover, it is currently unknown how many of these buffer genes exist, to what extent they differ in their mechanistic principles and buffering power, and whether they may have different evolutionary implications as a result.

It is important to highlight that the concept of buffer genes has been a subject of intense debate within the scientific community. Some argue that the notion of buffer genes may be an oversimplification or even a misinterpretation of complex genetic interactions. Central to this debate is the argument that deleting or reducing the function of any gene could potentially reveal hidden genotypic variation. This suggests that an increase in phenotypic variance following environmental or genetic changes might be a widespread occurrence, particularly in traits influenced by gene-environment interactions or epistasis (Siegal and Bergman, 2002; Hermisson and Wagner, 2004). The other point of debate is whether the release of cryptic genetic variation, following reduced activity of so-called buffer genes, should be taken as proof of the existence of these buffer genes. Critics argue that observing phenotypic changes upon the perturbation of a gene does not conclusively prove that the gene’s primary function is to buffer genetic variation. Rather, it may indicate a more complex interplay within the gene regulatory network that is not solely dedicated to buffering (Hermisson and Wagner, 2004; Geiler-Samerotte et al, 2016). Moreover, the debate extends to whether or not the buffering capacity of genes is the result of adaptive evolution, with buffer genes having evolved to allow the accumulation of hidden genetic variation that can be released in times of stress (e.g., environmental changes) (Rutherford, 2003).

One confounding factor in the discussion surrounding the existence and action of buffer genes is the absence of clear definitions and uniform nomenclature (Wagner, 2012; Masel and Bergman, 2003; Riederer et al, 2022). Throughout this review, the term “mutational robustness” is used broadly, referring to the extent to which a phenotype remains invariant in spite of changes in genotype, including both standing genetic variation and/or de novo mutations, and “evolvability” is used to refer to the capacity of a biological system to generate adaptive, heritable phenotypic variation (Fares, 2015; Masel and Bergman, 2003; Masel and Siegal, 2009). We define buffer genes as genes whose activity, in one way or another, contributes to mutational robustness by influencing the phenotypic outcome of standing genetic variation and/or de novo mutations. Given that genetic interactions are ubiquitous, the former definition could imply that any gene is a buffer gene since the activity of any gene likely shows some level of interaction with a set of alleles or specific mutations. We therefore only consider a gene to be a buffer gene when its activity influences the phenotypic outcome of an exceptionally broad subset of alleles or mutations. Importantly, our definition of a buffer gene does not assume that its buffering capacity has been selected for that specific function; it is possible that the buffering is merely a by-product of the gene’s primary function, causing an exceptional level of genetic interactions. We hypothesize that mutational buffering can occur on different levels, both at the level of gene expression (indirect) or of the protein itself (more direct).

In this review, we provide an overview of the known mechanisms that confer mutational robustness and the role of mutational robustness in preserving phenotypic stability in the face of standing genetic variation or de novo mutations. We focus on the most studied mechanism, using the chaperone *HSP90* as a key example and discuss the evidence for its influence on the manifestation of the phenotypic effects of cryptic standing genetic variation and de novo mutations. Finally, we explore the balance between mutational robustness and evolvability, shedding light on why and how such mechanisms of mutational robustness exist.

## Mechanisms of mutational robustness

The scientific literature does not provide a rigorous categorization of mutational robustness mechanisms. Instead, mutational robustness is presented as an interplay of different factors.

Some mechanisms include DNA methylation and chromatin remodeling, which can act as a buffer against genetic variation by stabilizing gene expression and minimizing the effects of genetic variation or mutations (Besselink et al, 2023; Hummel et al, 2017). For example, a study showed the significant role of chromatin regulators in buffering gene expression diversity between closely related species (Tirosh et al, 2010). In this study, eight regulators were individually deleted in two closely related yeast species, *Saccharomyces cerevisiae* (budding yeast) and *Saccharomyces paradoxus*. The study revealed that deleting these regulators generally increased the expression differences between the two species, suggesting a role for the regulators in buffering expression variation (Tirosh et al, 2010).

Furthermore, an RNAi screen in *Caenorhabditis elegans* (round worm) showed that the inactivation of chromatin-modifier hub genes enhances the phenotypic consequences of mutations (Lehner et al, 2006). Together, these results suggest that regulatory genes could modulate the effect of mutations by allowing organisms to adapt the expression of other genes to mitigate the effects of mutations.

Other mechanisms of mutational robustness include (i) genetic redundancy and (ii) buffer genes. Genetic redundancy refers to the existence of genes or pathways that can compensate for the malfunction of a certain (mutated) gene or pathway (Li et al, 2010; Kafri et al, 2006; Kuzmin et al, 2022; Van de Zande et al, 2023). An obvious example of genetic redundancy is diplo/polyploidy. While genetic redundancy primarily focuses on how one gene copy compensates for the function of the other copy in the event of a loss-of-function mutation, buffer genes are postulated to interact with a broader range of mutations or alleles across the genome (Queitsch et al, 2002; Rutherford and Lindquist, 1998; Jarosz and Lindquist, 2010). Most suggested mechanisms for how buffer genes mitigate the effect of multiple mutations or alleles involve molecular chaperones. Chaperones help proteins fold and function correctly, even if their primary sequence is mutated. In this way, chaperones can allow a mutated protein to function correctly and therefore reduce the phenotypic consequences of mutations that affect a protein’s structure (Fares et al, 2002; Maisnier-Patin et al, 2005; Tokuriki and Tawfik, 2009; Tokuriki et al, 2008; Queitsch et al, 2002). Importantly, however, the buffering capacity of chaperones does not appear to be limited to their direct ability to help mutated proteins fold correctly. A recent study showed that the specific chaperone Hsp90 can also buffer mutations in regulatory regions, likely because it interacts with numerous proteins involved in gene regulation (Jakobson et al, 2023) (see further).

**Figure 2:**
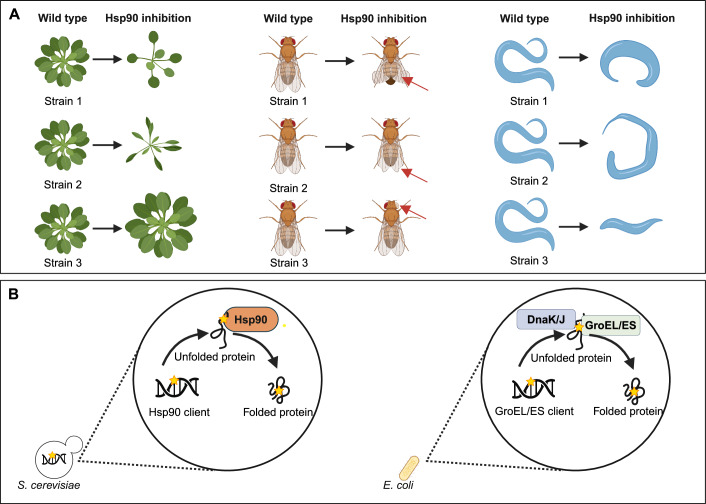
Phenotypic consequences of Hsp90 impairment and proposed buffering mechanisms. (**A**) Phenotypic consequences of Hsp90 impairment in model organisms: diverse phenotypes associated with Hsp90 impairment in *A. thaliana*, *D. melanogaster*, and *C. elegans* (Fig. 2A for *A. thaliana* is inspired by Figs. 1 and 2 in (Queitsch et al, 2002), *D. melanogaster* is inspired by Fig. 1 in (Rutherford and Lindquist, 1998), *C. elegans* is inspired by Fig. 1 in (Burga et al, 2011)). (**B**) Suggested buffering mechanism: One of the proposed mechanisms by which chaperones like Hsp90, GroEL/ES, and DnaK provide robustness is by ensuring the correct folding of their client proteins even in the presence of mutations (Fig. 2B is a conceptual figure to illustrate the proposed mechanisms of buffering, including direct Hsp90-client interaction (Zabinsky et al, 2019), as well as interactions involving GroEL/ES, or DnaK/J with their respective clients (Fares et al, 2002; Tokuriki and Tawfik, 2009; Aguilar-Rodríguez et al, 2016)). However, one outstanding question that remains at least partly unanswered is to what extent these chaperones can also buffer non-client proteins and, if so, how this buffering occurs. Colored stars in the cell’s DNA represent mutations. Figure created with BioRender.com.

The best-studied example to date of a mutational buffer protein in eukaryotes is the chaperone Hsp90, which has been shown to suppress the effect of cryptic genetic variation in many different organisms (e.g., *Arabidopsis thaliana* (thale cress), *S. cerevisiae*, *Drosophila melanogaster* (fruit fly), and *C. elegans* (Fig. 2A) (Rutherford and Lindquist, 1998; Lempe et al, 2013; Jarosz and Lindquist, 2010; Burga et al, 2011).

Previous studies in bacteria have also highlighted the role of chaperones in providing mutational robustness, and even suggested that the levels of these chaperones might adapt over time to help counterbalance the effects of de novo mutations (Fig. 2B). For instance, mutation-accumulation experiments (i.e., experiments where populations are periodically bottlenecked so that mutations accumulate largely independent of selection) in bacteria found that levels of the *dnaK* (*HSP70*) and *groEL* (*HSP60*) chaperones were increased in cells with high mutation loads (Maisnier-Patin et al, 2005). In addition, *groEL* overexpression could improve the fitness of these mutant cells, but not of ancestral wild-type cells, presumably by masking the effect of deleterious mutations (Maisnier-Patin et al, 2005; Fares et al, 2002). Moreover, when looking at the genetic variation present in proteins depending on GroEL for proper folding, it was found that these proteins have a higher ratio of non-synonymous to synonymous mutations compared to proteins that fold independently of GroEL; suggesting that buffering of (deleterious) mutations by GroEL can increase the evolutionary rate of its clients (Williams and Fares, 2010). In another study, researchers explored the buffering role of the DnaK system in *Mycobacterium smegmatis* (Fay et al, 2021). *M. smegmatis* cells with a mutation in the RNA polymerase gene *rpoB* gene, conferring resistance to rifampin, showed a significant fitness defect when the DnaK cochaperone DnaJ was absent. These findings indicate that the DnaK system might directly stabilize the mutant RNA polymerase, thereby enhancing rifampin resistance.

Apart from *HSP90* and chaperones, other genes have been identified that, when mutated or impaired, reveal cryptic genetic variability. For instance (Lemus et al, 2023), identified *AGO1* in *A. thaliana* as a key buffer gene: hypomorphic mutations in *AGO1* can uncover previously hidden genetic variations, which in turn affect critical traits such as the timing of flowering and leaf development. *AGO1* appears to help maintain stability of these traits via miRNAs (small RNA molecules that control gene expression). Importantly, while Ago1 physically interacts with Hsp90, Ago1-buffered loci did not overlap with those buffered by Hsp90 (Levy and Siegal, 2008). Moving forward, we will focus on *HSP90* as the key example of a buffer gene.

## The multifaceted roles of *HSP90* in genetic buffering

*HSP90* is a highly conserved gene throughout eukaryotic lineages and arguably stands as the most-studied example of a buffer gene. Hsp90’s prime function is assisting in the proper folding and maturation of client proteins and ensuring their function, stability, and preventing protein misfolding and aggregation (Fig. 2B) (Jackson, 2013; Yan et al, 2010; Caplan et al, 2003). In the next paragraphs, we summarize the current knowledge about the buffering role of Hsp90 and use it as an example to introduce some key aspects and questions regarding buffer genes. For simplicity throughout this review, we will use “*HSP90*” when referring to the gene and “Hsp90” when referring to the protein, regardless of the organism being discussed.

Seminal work in *D. melanogaster* showed that Hsp90 can influence the effect of standing genetic variation (Rutherford and Lindquist, 1998). Impairing Hsp90 function, either via drug-based inhibition or specific mutations, results in diverse phenotypes that depend on the genetic background of the fly, e.g., eye defects in one strain and wing defects in another (Fig. 2A). Similarly, studies in animals, plants, and yeast confirmed that reduced Hsp90 activity results in a wide range of heritable phenotypes, indicating that Hsp90 affects the consequences of standing genetic variation in many species (Queitsch et al, 2002; Jarosz and Lindquist, 2010; Burga et al, 2011; Cowen and Lindquist, 2005; Chen and Wagner, 2012; Casanueva et al, 2012; Yeyati et al, 2007; Iyengar and Wagner, 2022b).

Hsp90’s interaction with a broad range of genes implies that its buffering capacity is complex and could also act at the level of gene regulation. A recent study explored the role of Hsp90 in influencing gene expression across five highly diverged *S. cerevisiae* strains by analyzing the transcriptome in the presence and absence of the Hsp90 chemical inhibitor geldanamycin. Hung et al found that a significant number of genes showed differential expression depending on the strain and the activity of Hsp90, implying that individual strains can have distinct gene expression responses when Hsp90’s activity is compromised. Furthermore, this study also identified key transcription factors that seem to play a crucial role in driving this expression variability. Upon Hsp90 inhibition or under environmental stress, the activity of these Hsp90-dependent transcription factors differs among strains, leading to strain-specific expression of their target genes. These findings highlight the potential widespread evolutionary implications of (differences in) Hsp90-dependent variations in natural yeast populations, since these variations provide a large repertoire for natural selection to act on (Hung et al, 2023).

Expanding on this, Jakobsen and coworkers found that Hsp90 can buffer standing genetic variation in numerous natural coding and non-coding regions of the yeast genome (Jakobson et al, 2023), with variation in non-coding regions accounting for the largest proportion of Hsp90-dependent buffered variants. Hsp90’s most pronounced effects were on regulatory variants situated upstream of the transcription start site of genes, suggesting a potential role for Hsp90 in modulating the recruitment of transcription factors or in transcription initiation. Moreover, the study also identified variants in the untranslated regions of genes as part of Hsp90-dependent buffered variants, suggesting that Hsp90 also plays a role in post-transcriptional regulation.

Hsp90’s capacity to buffer cis-regulatory variations might be linked to its interaction with proteins from three key groups: kinases (which phosphorylate other proteins), transcription factors (which facilitate the transcription of DNA to RNA), and E3 ubiquitin ligases (which mark proteins for degradation). The study’s findings suggest that the targets of specific kinases and transcription factors, which are known to interact with Hsp90, were more likely to have their phenotypic outcomes modified by Hsp90’s activity. Overall, this study highlights Hsp90’s complex role in genetic regulation, showing that it can mitigate the effects of mutations not only through direct interactions with specific client proteins but also through a wider network of regulatory proteins, affecting a broad spectrum of genetic variations. Furthermore, the interactions between Hsp90 and these genetic variants were often found to be condition-specific, underscoring its importance in adaptation, specifically in rapidly changing environments (Jakobson et al, 2023).

Hsp90 buffering might also extend to non-genetic variation. Specifically, Hsieh and colleagues showed that reduced Hsp90 levels result in morphological heterogeneity in an isogenic yeast population. This morphological heterogeneity is caused by lower levels of the Cla4 protein, a regulator of septin formation. Reduction of Hsp90 activity destabilizes Cla4; with ~40–60% of cells with lower Cla4 activity displaying filamentous growth. This morphological switch was also observed in other yeast species. Exactly how reduced Hsp90 levels lead to reduced Cla4 stability is still unclear, although genetic and physical interactions between Hsp90 and Cla4 suggest that Cla4 could be a Hsp90 client (Zhao et al, 2005; Hsieh et al, 2013).

Whereas most studies focus on the interaction between Hsp90 and standing genetic variation, Hsp90 might also modulate the phenotypic outcome of certain de novo mutations. For example, natural, stochastic variation in the expression levels of *HSP90* influences the phenotypic effect of a mutation that can in some cases lead to developmental disorders in the worm *C. elegans* (Burga et al, 2011). In this organism, T-box transcription factor *tbx-9* null mutants cause an incompletely penetrant defect in larval morphology due to abnormal development of the epidermis and muscle, with around 70% of embryos displaying abnormal morphology with low *HSP90* expression levels. Notably, for individuals expressing high levels of *HSP90*, the negative effects of the *tbx-9* mutation are significantly mitigated (Burga et al, 2011).

Another example of the interaction between Hsp90 and de novo mutations was provided by Cowen and Lindquist (Cowen and Lindquist, 2005). They show how lowering Hsp90 activity reduces the number of spontaneous drug-resistant mutants obtained after exposing large populations of *Candida albicans* to the antifungal drug fluconazole. Interestingly, they also show that drug-resistant mutants that emerged in a patient suffering from candidiasis also depend on high Hsp90 activity. At first sight, one might expect that Hsp90’s buffering capacity would reduce the phenotypic effect of mutations and therefore reduce evolvability. Paradoxically, however, in this case Hsp90 activity appears to potentiate new mutations to have immediate phenotypic consequences.

Studies have proposed that the molecular chaperone Hsp90 can also act as a capacitor that promotes the maintenance of genetic variation in a population by reducing the -mostly deleterious- phenotypic effects of mutations (Rutherford and Lindquist, 1998; Queitsch et al, 2002; Sangster et al, 2004). This would enable stabilized or buffered genetic variants to accumulate without (strong) phenotypic consequences; allowing for hidden or so-called “cryptic genetic variation” to build up and persist in a population (Sangster et al, 2008; Zabinsky et al, 2019). Reducing the activity of Hsp90 through heat stress or pharmacological inhibition reveals cryptic genetic variation. This unmasking of phenotypic variation in traits is heritable and can give rise to new adaptive traits within natural populations (Rohner et al, 2013; Rutherford and Lindquist, 1998; Queitsch et al, 2002).

Moreover, the role of Hsp90 as a potentiator of genetic variation has been observed in several studies (Jarosz, 2016; Lindquist, 2009; Zabinsky et al, 2019; Cowen and Lindquist, 2005; Jarosz and Lindquist, 2010). It is suggested that Hsp90’s ability to fold inherently unstable protein variants enables specific genetic variations to quickly form new phenotypes. If this protein-folding ability is compromised, the mutated protein might not fold correctly and the newly created traits may be lost (Jarosz and Lindquist, 2010). A prime example of Hsp90 acting as a potentiator is deoxycholate resistance in *S. cerevisiae*. A yeast strain with a specific allele of the transcription factor gene *PDR8* shows resistance to deoxycholate. Notably, Hsp90 inhibition caused strains carrying this *PDR8* allele to become deoxycholate sensitive. Investigation of other Pdr8-dependent phenotypes revealed that these were not affected upon Hsp90 inhibition; indicating that Pdr8 does not require Hsp90 function. Instead, this Pdr8 variant likely is resistant to deoxycholate because of Hsp90’s interaction with a yet unknown gene that is both important for deoxycholate resistance and interacting with Pdr8 (Jarosz and Lindquist, 2010). Another example of the complex interactions that may underlie the role of Hsp90 as a potentiator of antibiotic resistance was reported in (Cowen and Lindquist, 2005). Here, Hsp90’s effect does not depend on its chaperoning activity of the mutated proteins that confer (fluconazole) resistance. Instead, Hsp90 chaperones calcineurin, a regulatory protein known to regulate the response to azoles. In this way, Hsp90 could potentiate the effect of different mutants on fluconazole resistance via a common regulator (Cowen and Lindquist, 2005).

In summary, the “capacitor” effect of Hsp90 refers to its role in buffering the effects of mutations so that they become near-neutral and can therefore accumulate and persist in a genome, while they may prove adaptive when the environment changes. The “potentiator” effect involves the capacity of Hsp90 to influence the effect of mutations, thereby ensuring that some mutations have a beneficial effect rather than being negative. Importantly, some authors are not particularly fond of introducing terms like “capacitor”, “potentiator”, or “buffering”. Instead, they prefer the term “epistasis”, believing it more precisely and simply describes the interaction between Hsp90 and mutations. They also argue that both “capacitor” and “potentiator” are specific forms of epistasis that involve conditional neutrality, rather than being separate concepts (Siegal and Leu, 2014; Geiler-Samerotte et al, 2016). Please note that the way we define “buffer genes” in this review includes both the “capacitator” and “potentiator” effect, which we believe is a more clear, all-encompassing way to describe how certain genes can alter/influence the phenotypic effect of genetic variation.

The potential buffering role of Hsp90 has also received criticism. Inhibiting Hsp90 can cause genomic instability, e.g., an increase of point mutations, insertions/deletions, microsatellite slippage events, somatic homologous recombination, and transposon activity which could influence an organism’s fitness (Zabinsky et al, 2019). Consequently, this genetic instability could be responsible for the phenotypic variation upon Hsp90 inhibition, rather than being a consequence of Hsp90’s capacity to buffer genetic variation. However, other studies demonstrated that both drug and mild temperature treatments to inhibit or overwhelm Hsp90 consistently yield the same phenotypes in organisms with different genetic backgrounds (Kaplan and Li, 2012; Zabinsky et al, 2019). This uniformity is rather unlikely to be formed by new mutations resulting from genomic instability and more likely suggests a link with the unmasking of pre-existing, standing genetic variation (Zabinsky et al, 2019; Kaplan and Li, 2012).

## The potential of Hsp90 in disease treatment

The buffering capacity of Hsp90 might also play a role in various genetic diseases. For example, cancer cells typically show higher mutation rates, which has led to the hypothesis that these cells rely on Hsp90’s ability to mitigate the negative phenotypic consequences of mutations (Workman, 2004; Jarosz, 2016). Interestingly, a recent study found that tumors with a large number of (somatic) mutations show upregulation of genes involved in degrading and refolding proteins, such as *HSP90* (Tilk et al, 2023). The hypothesis is that tumors upregulate the expression of chaperones to fold mutated proteins so that these proteins retain their normal function, similar to what is observed in yeast, bacteria, and viruses (Elena et al, 2006; Bobula et al, 2006; Fares et al, 2002; Maisnier-Patin et al, 2005). These findings highlight the potential of chaperone inhibitors in cancer therapy.

The clinical use of Hsp90 inhibitors has been explored as a cancer treatment strategy for over two decades (Baaklini et al, 2020; Neckers and Workman, 2012; Kim et al, 2009; Whitesell and Lindquist, 2005; Taipale et al, 2010; Xiao and Liu, 2019; Tomala and Korona, 2008). However, this strategy is highly contested, since inhibiting Hsp90 may have unwanted side effects. First, Hsp90 is the ‘hub of hubs,’ which means it interacts with a vast number of proteins and can influence almost every regulatory pathway in the cell. Thus, we need a better understanding of exactly how Hsp90 rewires signaling pathways (Zabinsky et al, 2019; Jarosz, 2016). Second, Hsp90 plays an essential role in affecting epigenetic changes and in the generation of new genetic variations by its role in chaperoning many proteins functioning in various DNA maintenance and repair pathways (Wong and Houry, 2006; Jarosz, 2016; Zabinsky et al, 2019). Lastly, the effects of Hsp90 inhibition on the evolution of new traits, such as drug resistance, remain uncertain (Tilk et al, 2023; Jarosz, 2016). Despite these challenges, researchers persist in their efforts to optimize Hsp90 inhibitors and to develop strategies that maximize their potential while addressing their drawbacks. Approaches under exploration include combining Hsp90 inhibitors with other treatments, creating isoform-specific inhibitors, and tailoring therapies based on a patient’s molecular profile.

## Mutational robustness and cryptic genetic variation

Cryptic genetic variation is a type of genetic variation that remains phenotypically neutral under normal conditions, but can become phenotypically active with environmental or genetic changes, including the impairment of buffer gene activity (Rutherford and Lindquist, 1998; True and Lindquist, 2000; Masel, 2006; Jarosz and Lindquist, 2010; Hansen et al, 2023). Cryptic genetic variation has been suggested to facilitate adaptation to different environments and hence facilitate evolvability (Payne and Wagner, 2019). First, cryptic genetic variation that is revealed upon environmental, physiological, or genetic perturbation (and more generally the loss of mutational robustness), could prove to be adaptive. This has indeed been observed in *S. cerevisiae* and *A. thaliana* (Sangster et al, 2008; Jarosz and Lindquist, 2010). Specifically, for *S. cerevisiae*, inhibiting Hsp90 across genetically diverse strains and across diverse conditions resulted in substantial changes in growth under specific conditions. Importantly, these growth changes were sometimes positive and sometimes negative, supporting the hypothesis that (part of) cryptic genetic variation can be adaptive under some conditions (Jarosz and Lindquist, 2010). Second, cryptic genetic variation facilitates different genetic backgrounds to arise. In case a new mutation arises that shows interaction with some cryptic variants, it can have different effects in the different backgrounds, thereby increasing phenotypic diversity on which selection can act (Paaby and Rockman, 2014).

When reducing a gene’s activity leads to the activation of cryptic genetic variation (in other words, if a (change in) phenotype can be observed), studies have often taken this as a sign of reduced mutational robustness, implying that the impaired gene is a mutational buffer gene. However, this interpretation has been heavily debated, with researchers arguing that the revelation of cryptic genetic variation should not be taken as evidence for mutational robustness against all types of mutations. Instead, cryptic genetic variation could represent mutations (alleles) that survived the filter of natural selection to exist in their cryptic form; and are hence different from random, new mutations (Geiler-Samerotte et al, 2016; Richardson et al, 2013; Paaby and Rockman, 2014; Hermisson and Wagner, 2004). In other words, while decreasing robustness will indeed reveal cryptic genetic variation, the converse (revelation of cryptic genetic variation proves that robustness has decreased) is not necessarily correct.

As mentioned above, so far, only a handful of studies have investigated mutational robustness to de novo mutations (Geiler-Samerotte et al, 2016; Fares et al, 2002; Richardson et al, 2013; Aguilar-Rodríguez et al, 2016; Cowen and Lindquist, 2005; Burga et al, 2011). Moreover, while these studies were the first to investigate mutational robustness to de novo mutations and provided crucial insights, they do focus on a specific buffer gene product (Hsp90 in one case and Htz1 in the other case) and how it influences the phenotypic outcome of spontaneous, unselected mutations (Geiler-Samerotte et al, 2016; Richardson et al, 2013). Hence, we currently do not know if and how these results hold for other candidate buffer genes. For example, a study in *E. coli* showed that the chaperon GroEL/ES can provide mutational robustness to de novo mutations that were introduced by multiple rounds of direct mutagenesis to certain genes (Tokuriki and Tawfik, 2009). When *groEL/ES* was overexpressed, there was a higher accumulation of mutations in the tested genes, suggesting that chaperones allowed for a broader range of mutations to be tolerated. Furthermore, enzyme variants with more destabilizing mutations displayed higher enzymatic activity in the presence of *groEL/ES* overexpression, suggesting that GroEL/ES buffers the effect of both destabilizing and adaptive mutations (Tokuriki and Tawfik, 2009). However, a study in *S. cerevisiae* suggested that Hsp90 does not necessarily buffer the effect of de novo mutations (Geiler-Samerotte et al, 2016). Instead, natural selection preferentially allows buffered alleles to persist in populations and thereby creates the false impression that Hsp90 confers greater robustness. Specifically, researchers used a collection of yeast strains from a mutation accumulation experiment, hypothesizing that the mutations in these strains represent de novo mutations accumulating under no or reduced selection. As a control, they used strains isolated from natural environments, hypothesizing that these strains had already undergone selection. Similar to previous studies, they found that Hsp90 does buffer the standing genetic variation in natural strains. However, in strains with de novo mutations, inhibiting Hsp90 led to a decrease in phenotypic variation. The study concluded that natural selection makes Hsp90 appear as a buffer by preferentially enriching buffered alleles; while for de novo mutations, Hsp90 acts more often as a potentiator instead of a buffering capacitor (Geiler-Samerotte et al, 2016).

Taken together, the interaction between de novo mutations and buffer genes is complex and contested. A comprehensive, multidisciplinary approach is essential to untangle this complexity further (Box 1).

Box 1 In need of answers Despite intense theoretical and experimental research into the phenomenon of mutational robustness and the role of buffer genes, several seminal questions remain at least partly unanswered.

1. How general is the phenomenon of mutational robustness? Is it specific to a limited set of proteins, or is it common to many genes? In other words, how unique is *HSP90*? What are the specific characteristics of a buffer gene?
2. Are organisms robust to the effect of de novo mutations, and do some buffer genes contribute to this robustness?
3. Do buffer genes buffer the effect of a broad range of mutations, or do different genes mitigate the effect of different types of mutations?
4. Are some buffer genes more important in certain conditions or genetic backgrounds than others?
5. What are the molecular mechanisms behind mutational robustness?
6. What are the evolutionary implications of robustness? How does it shape the survival and adaptation of organisms in different environments? How does it influence the pace of adaptation and its trajectories?

## The role of mutational robustness in evolvability

**Figure 3:**
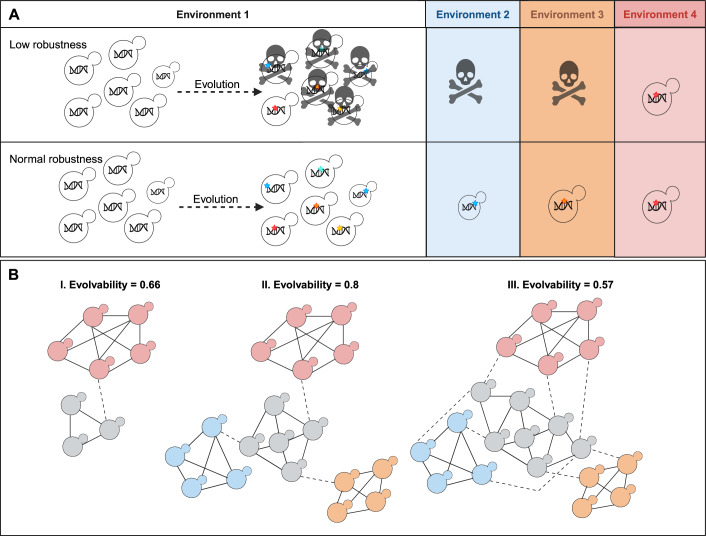
Mutational robustness and evolvability: capacitor effect. (**A**) The capacitor effect: the link between mutational robustness and evolvability in a population. Most spontaneous mutations are deleterious. This implies that in populations with low mutational robustness, these mutations will be removed from the population through natural selection, leading to a decline in standing genetic variation. This reduced genetic variation ultimately lowers the population’s capacity for evolutionary adaptation. In contrast, normal robustness buffers the fitness effect of mutations, resulting in a higher number of near-neutral mutants that survive the bottleneck of selection and contribute to the buildup of standing genetic variation that can ultimately help the population adapt to new conditions. This effect is sometimes referred to as the “capacitor effect” of buffer genes. Colored stars in the yeast cell’s DNA represent mutations. This representation is purely conceptual and made to simplify the concept of the capacitor effect of a buffer gene. (**B**) Limits to the capacitor effect: neutral genotypic networks are those in which several genotypes (here depicted as yeast cells) are connected through single mutations and lead to the same phenotype (phenotype is symbolized by the color of the yeast cell). A simplified suggested method to measure evolvability is to calculate evolvability as the ratio between accessible phenotypes and the number of genotypes in a particular network. A small genotypic network (I) has a low potential to evolve novel phenotypes. For example, in I there are two phenotypes accessible (red and grey) and three genotypes in the grey network, resulting in an evolvability ratio of 0.66 (2 phenotypes divided by 3 genotypes) for the grey population. As the network increases in size (II), the number of phenotypes that are accessible via single mutations also increases, resulting in increased evolvability. For example, the evolvability of the grey subpopulation in network II has increased to 0.8 (4 phenotypes divided by 5 genotypes) by adding two additional genotypes to the network, which in turn resulted in a total of 4 accessible phenotypes. A further increase of the mutational genotypic network, such as in genotype (III), decreases evolvability (network III has decreased its evolvability to 0.57 (4 phenotypes divided by 7 genotypes) because the extra genotypes start showing increased overlap in the phenotypes they give access to (figure inspired by Fig. 1 in (Fares, 2015)). Figure created with BioRender.com.

How might mutational robustness influence the ability of a population to produce adaptive variation? At first sight, it may appear that mutational robustness diminishes evolvability, since it masks or limits the phenotypic effects of mutations and thus also “hides” them from natural selection, reducing the supply of beneficial mutations that fuel adaptation (Masel, 2006; Lenski et al, 2006; Hansen et al, 2023). However, as already indicated above, mutational robustness might enhance evolvability in the longer term, by allowing populations to accumulate mutations as (near-) neutral genetic variants that in the absence of buffering would have severe negative fitness effects and would therefore have been lost (Fig. 3A) (Hansen et al, 2023; Jakobson et al, 2023). This implies that the buffer genes that contribute to increased mutational robustness may also contribute to increased standing genetic variation. This effect can impact evolution in 3 non-mutually exclusive and intertwined ways. (i) The increased genetic variation reduces the mutational distance towards potentially adaptive phenotypes by occupying a broader proportion of the “genotype network” (Fig. 3B) (Hansen et al, 2023; Wagner, 2007). Having more genotypes in a population increases the chances of a highly fit genotype only being one or a few mutational steps away (Wagner, 2012). (ii) The increased genetic variation provides a broader range of genetic backgrounds within which new mutations occur. If there is epistasis, this can allow the population to have access to a broader range of evolutionary solutions, known as exaptation (Paaby and Rockman, 2014). (iii) Accumulated neutral, cryptic genetic variation may be revealed to selection when conditions change, e.g., under environmental stress or as a consequence of reduced buffering activity. It is possible that the originally negative mutation is now adaptive, e.g., because of the changed condition or due to other mutations that have since accumulated in the same genome (Wagner, 2012, 2011, 2005). However, it is also important to note that extreme genetic robustness might in fact have the opposite effect and lead to lower evolvability (Fares, 2015) (Fig. 3B, case III).

*Astyanax mexicanus* (Mexican cavefish) is a concrete example to illustrate these concepts. This species is a striking example of how buffer genes and cryptic genetic variation have interacted to influence evolvability (Rohner et al, 2013). *A. mexicanus* is a species that consists of both surface-dwelling and cave-dwelling populations. The cave-dwelling populations have evolved over time to lose their functional eyes. In the dark, where vision is impossible, reduced eye size represents a beneficial trait in terms of energy cost. Rohner and colleagues explored whether such a trait could be cryptic and under the control of Hsp90. To investigate this, they chemically inhibited Hsp90 in cavefish embryos. This perturbation led to the development of cavefish with larger or smaller eyes and eye sockets, which is consistent with the hypothesis that Hsp90 masks cryptic genetic variation. Furthermore, when the fish with smaller eyes were bred, their offspring also had smaller eyes, confirming the genetic basis of this trait. Additionally, this study investigated the environmental stressor that could release such variation and revealed that underground water has significantly lower salinity than surface water. Notably, growing cavefish embryos in low-salinity water yielded a wider eye size variation, providing substrates for natural selection to work on and ultimately allowing natural selection to select for cavefish with no functional eyes. This example suggests that Hsp90 activity not only influences the exposure of cryptic genetic variation, but might also play a prominent role in the evolution of adaptive traits (Rohner et al, 2013).

Moreover, several studies have linked mutational robustness to increased evolvability (Jiang et al, 2023; Bloom et al, 2007; Draghi et al, 2010; Mcbride et al, 2008; Lauring et al, 2013; Tokuriki and Tawfik, 2009; Rigato and Fusco, 2016; Zheng et al, 2019). For example, a study on the lytic RNA bacteriophage φ6 showed a positive correlation between mutational robustness and evolvability (Mcbride et al, 2008). In the context of viruses, mutational robustness implies that the virus is still able to infect and replicate in spite of mutations. In this study, two populations of viruses with high and low robustness were evolved. These two levels of robustness were developed by serially passaging viral populations through the bacterium *Pseudomonas syringae* under conditions where bacteria were either infected by a single virus (low coinfection) or simultaneously by multiple viruses (high coinfection). The idea is that high coinfection helps a defective virus particle reproduce, because defective proteins are complemented by the production of the same functional protein by another virion that is present due to the coinfection, thus allowing less-robust virus strains to thrive. Viruses with high levels of coinfection showed a greater variance in fitness, indicative of reduced mutational robustness, while low coinfection viruses showed the opposite. Subsequent sequencing of these evolved populations showed the robust population (low coinfection) had accumulated more mutations compared to the non-robust population. Moreover, to explore the link between robustness and evolvability, they exposed the evolved populations to direct heat shock. Robust populations showed higher survival rates compared to the non-robust populations, indicating that mutational robustness promotes evolvability in a new environment (in this specific case, a heat shock environment) (Mcbride et al, 2008). However, the exact molecular mechanism underlying the robustness is not known, making it difficult to interpret the results.

Other studies have attempted to explore the impact of tuning the expression of buffer genes on adaptation dynamics (Aguilar-Rodríguez et al, 2016; Iyengar and Wagner, 2022a; Lukačišinová et al, 2020). For example, a mutation accumulation experiment followed by whole-genome resequencing investigated the effects of *dnaK* (*Hsp70*) overexpression on the ability of *E. coli* cells to deal with accumulating mutations. The findings revealed that lines of *E. coli* overexpressing *dnaK* survived longer (and for more generations) compared to lines not overexpressing *dnaK*. This could be an indication of the role of DnaK in buffering accumulating deleterious mutations (Aguilar-Rodríguez et al, 2016). Expanding on this, the authors conducted a comparative genomic analysis to evaluate whether proteins that are strong clients of DnaK evolve at distinct rates compared to non-client proteins. This analysis covered natural populations of *E. coli*, *Salmonella enterica*, and other gamma-proteobacterial species. The results indicated that genes that interact strongly with DnaK tend to evolve faster compared to genes that interact weakly with DnaK, again suggesting an interplay between the buffer gene and the evolvability of its respective client proteins (Aguilar-Rodríguez et al, 2016).

Interestingly, a recent study investigated the role of mutational robustness in gene evolution (Jakobson et al, 2023). The results suggest that sequence variation in evolutionary young genes, and particularly variations in the cis-regulatory regions of these genes, were most affected by Hsp90 activity. As such, fluctuations in the buffering effect of Hsp90 create a significant source of new and diverse traits that may provide evolutionary selectable variation. This is especially true for genes that are important for adapting to specific environmental conditions (niche-specific adaptation) and that have alleles causing significant changes in response to different conditions (Jakobson et al, 2023).

Although most empirical work has shown that robustness can enhance evolvability under certain circumstances, theoretical studies suggest that this relationship may not always be straightforward. Specifically, evolutionary models show that robustness may either enhance or diminish evolvability, depending on e.g., the complexity of the environment, mutation rate, population size, and the type of robustness (Lenski et al, 2006; Wagner, 2007; Masel and Trotter, 2010). For example, a recent study used computer simulations to model populations of multicellular organisms and eukaryotic cells, aiming to understand the impact of robustness on evolvability. The simulations mimicked real-life scenarios where organisms faced sudden environmental changes. The researchers used varying levels of robustness in the simulated organisms and observed how they evolved over time. The results suggest a sweet spot in robustness that maximizes evolvability, with organisms showing higher or lower robustness suffering a lower adaptation rate. Moreover, the researchers also observed an interesting dynamic pattern where populations with low robustness initially adapted more quickly to a new environment than those with high robustness. However, over longer periods, this trend was reversed (Jiang et al, 2023).

In summary, while robustness is pivotal for an organism’s evolutionary potential, the exact relationship between robustness and evolvability is complex. To understand this delicate balance, a focused and systematic approach that integrates both theory and experimental work is needed (Greenbury et al, 2016; Draghi and Ogbunugafor, 2023; Catalán et al, 2018; Draghi et al, 2010).

## Is mutational robustness itself a product of selection?

Importantly, whereas it is clear that some genes exert a certain level of buffering, the idea that such buffering represents a true example of canalization, i.e. a system that evolved with the specific aim of influencing or even controlling the expression of genetic variation and its effect on adaptive evolution is highly contested.

There are two main hypotheses in the field on the origin of mutational robustness (Eshel and Matessi, 1998; Kawecki, 2000; Omholt et al, 2000; Proulx and Phillips, 2005; Van Nimwegen et al, 1999; Wagner, 1996, 1998, 2000a; Wilke and Adami, 2003; Hansen et al, 2023). The first hypothesis suggests that mutational robustness is not an adaptive trait that has been directly selected for, but instead is an inherent property of biological systems (Wagner, 2005; Hermisson and Wagner, 2004). For instance, consider a typical network of interacting proteins. In such networks, most proteins have few connections, while a select few are extensively connected. This structure inherently provides robustness against disruptions, as the malfunction of a protein with fewer connections might have minimal impact due to its limited interactions (Siegal and Bergman, 2002). Thus, under this perspective, mutational robustness emerges as a byproduct of the system’s architecture and interactions, rather than being a trait that evolved for the purpose of enhancing robustness.

The second hypothesis suggests that mutational robustness is an adaptive trait that has evolved for at least two main reasons. Firstly, it has been proposed that mutational robustness evolved in response to mutations. Yet, several studies suggest that the development of mutational robustness as an adaptation to mutational stress is a rather unlikely scenario because mutations are rare and therefore do not cause (sufficient) selection pressure to increase robustness to mutations. However, theory predictions and experimental evolution propose that mutational robustness may evolve as a response to mutations under conditions where mutations occur frequently or in large populations (Wagner, 2005; Hansen et al, 2023; Wagner et al, 1997; Bloom et al, 2007). Secondly, mutational robustness may have evolved as an adaptation to environmental changes (Wagner, 2005; Milton et al, 2003). First, organisms are constantly encountering environmental and internal changes that could influence biological processes (Wagner, 2005; Milton et al, 2003). Second, genetic variation can influence organisms’ response to environmental change. For instance, an organism with the right genetic variation in a certain environment might provide an adaptive advantage. Therefore, over time, gene variants that are associated with robustness to these environments will become more prevalent in the population (Wagner, 2005; Milton et al, 2003).

Third, there is a clear association between the robustness to environmental changes and mutational robustness (Bloom et al, 2006; Butkovic et al, 2020; Hansen et al, 2023). For example, heat shock proteins such as Hsp90 help to fold proteins to protect against thermal noise but also buffer proteins against mutations (Félix and Wagner, 2008). Contrasting studies with *E. coli* found an intriguing anti-correlation between mutational robustness and environmental robustness (i.e., “the ability of an organism to maintain the same phenotype despite environmental noise during development”) after exposing the bacteria to genetic and environmental perturbations (Cooper et al, 2006; Stewart et al, 2012). The evidence suggests that while it is plausible that mutational robustness is selected under specific conditions, it is noteworthy that there is still a debate about how selection might shape such a trait (Scheiner and Lyman, 1991; Scheiner, 1993; Masel and Siegal, 2009). Hence, this relationship between mutational robustness and environmental robustness may not be straightforward and still require further investigation.

## Concluding remarks and future outlook

A certain level of mutational robustness is an inherent universal characteristic of living systems. However, there is still limited understanding of how it is accomplished at the molecular level and what the consequences of such phenomena are (Box 1). Moreover, some authors argue that buffering is merely an example of epistasis and complex genetic interactions for which no new term needs to be used (Siegal and Leu, 2014; Geiler-Samerotte et al, 2016). Although we agree with the idea that genetic buffering is a special example of epistasis and genetic interaction, we believe that the term buffer gene, to indicate a gene that affects the phenotypic outcome of an exceptionally broad subset of mutations or alleles, is a useful term to indicate interactions between a gene and various mutations or alleles.

Another point of debate is the role of cryptic genetic variation and whether its exposure should be taken as a marker for mutational robustness. Whereas decreasing robustness is indeed expected to reveal a certain extent of cryptic genetic variation, this should not be used as the sole criterion for identifying a gene as a buffer gene because the variation that is uncovered has already been exposed to selection and is therefore not a good representation of the variety of possible de novo mutations that can occur. As a consequence, it remains controversial whether buffer genes like *HSP90* also confer robustness to the broad, unbiased spectrum of de novo mutations (Hermisson and Wagner, 2004; Siegal and Bergman, 2002; Geiler-Samerotte et al, 2016) and the lack of strong experimental evidence for buffering against de novo mutations is sometimes used against the basic idea of mutational robustness and buffer genes.

Apart from the lack of studies that systematically investigate the relationship between buffer genes and de novo mutations, another important missing pillar in our understanding of mutational robustness is the lack of a clear metric. Most studies have primarily focused on measuring mutational robustness using fitness as a standard. While measuring fitness is an important and valuable approach, it alone is insufficient to grasp the complexity and intricacy of the phenomenon of mutational robustness. To achieve a comprehensive understanding of mutational robustness, future studies should implement various approaches, including molecular and genetic analysis, experimental evolution, various phenotypic read-outs, genomic analysis, examination of environmental variation, network analysis, and computational modeling.

Another gap in our understanding of mutational robustness is whether certain buffer genes are more critical under some specific conditions than others. A recent study suggests this might be the case, at least for Hsp90-dependent traits (Jakobson et al, 2023). For instance, while Hsp90 plays a significant role in cells growing in maltose, a type of sugar, its effect is negligible in raffinose, another type of sugar. This indicates that the effect of Hsp90 inhibition is highly specific to certain stress conditions (Jakobson et al, 2023). Thus, particular buffer genes may be more important in one condition than in another.

Taken together, the role of buffer genes in mutational robustness, evolvability, and disease is significant, but still poorly understood. The activity of buffer genes likely influences the accumulation of genetic variation, the outcome of disease-causing mutations, and ultimately influences if and how organisms can evolve and adapt to new environments. Hence, further identification and characterization of buffer genes may also open new avenues in medicine and biotechnology, where the activity of buffer genes may be tuned to increase the efficiency of adaptive laboratory evolution or breeding efforts. However, recent advancements in technology, such as cheap, high-throughput high-resolution sequencing, genetic mapping, and massive genome editing tools like CRISPR, coupled with AI tools like AlphaFold and comprehensive fitness analyses, enable us to address the knowledge gaps surrounding genetic buffering.
